# Contemporary evaluation and treatment of tricuspid regurgitation

**DOI:** 10.3389/fcvm.2024.1350536

**Published:** 2024-03-04

**Authors:** Andrei Minciunescu, Abbas Emaminia

**Affiliations:** Department of Cardiovascular Disease, Inova Schar Heart and Vascular, Falls Church, VA, United States

**Keywords:** tricuspid regurgitation, valvular heart disease, right-sided heart disease, heart failure, tricuspid valve

## Abstract

Valvular heart disease is a global health burden with substantial mortality. The left-sided valvular diseases have been extensively described using the robust treatment strategies available. By contrast, the right-sided diseases, particularly the tricuspid valve (TV) and associated regurgitation, still have much to be delineated. Worsening tricuspid regurgitation (TR) is associated with increased mortality; the non-invasive management is suboptimal; and surgical approaches carry significant risk. With advances in multimodality imaging, 3D echocardiography, improved understanding of TV anatomy, and pathophysiological mechanisms of primary and secondary regurgitation, as well as favorable data with transcatheter therapies, the field of TV management is rapidly evolving. This review aims to highlight pathophysiological mechanisms of TR, describe echocardiographic approaches to diagnosis and TV interrogation, and outline the latest transcatheter developments.

## Introduction

Valvular heart disease (VHD) remains a significant cardiovascular health burden in the United States and worldwide, with more than 33 million estimated global cases of rheumatic heart disease and 300,000 associated deaths (1). In the United States, an estimated 2.5% of the population carries a valvular heart disease diagnosis, and subsequent 25,000 deaths yearly from non-rheumatic VHD (2). Although the left-sided valvular disease has been well researched and reported, the right-sided pathology involving the tricuspid and pulmonic valves is a growing field of interest. Data suggest that clinically significant tricuspid regurgitation (TR) increases with age, and moderate or more TR is associated with twofold increase in hospitalizations and mortality compared with absent or mild TR (3, 4). Rapidly developing severe TR is an independent predictor of all-cause mortality and worsening heart failure (5–7), and population cohort analyses suggest that greater than 90% of patients remain untreated (8). Despite advances in TR diagnosis, guidelines have limited class I recommendations for surgical treatment owing to data paucity. Multimodality imaging, coupled with novel therapeutic strategies, allow for early, improved characterization and intervention.

## Etiology and natural history

The tricuspid valve (TV) is a uniquely complex structure, comprising a multiplanar asymmetric “D”-shaped annulus, a conventional configuration of three leaflets: anterior, posterior, and septal, of which the anterior leaflet is the largest, posterior, the shortest, and septal, the least mobile (9–11). The valve operates via an intricate arrangement of papillary muscles and chordae. The anterior papillary muscle attaches the anterior and posterior leaflets, while the posterior papillary muscle supports the posterior and septal leaflets (9). The chords may have greater than 20 insertions throughout the TV (9), and the corresponding choral anatomy has significant implications in transcatheter therapies.

**Figure 1:**
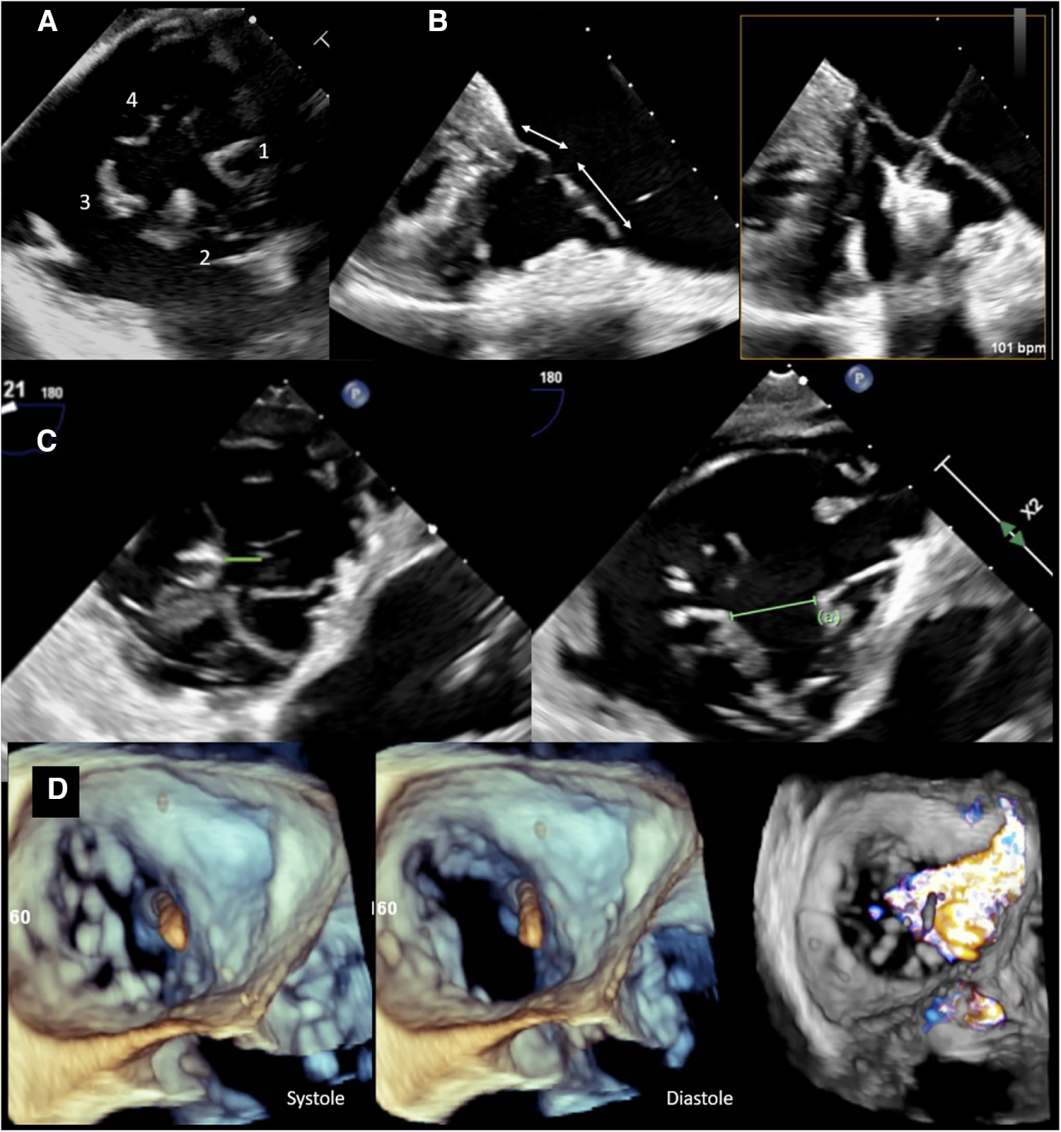
Preprocedural imaging of tricuspid valve. (**A**) Transesophageal view of a quadricuspid tricuspid valve with a (1) septal, (2) anterior, and (3,4) two posterior leaflets. (**B**) TEE view demonstrating septal and anterior tricuspid valve leaflets. There is sufficient length for TEER (>7 mm, left) on screening imaging, and during the procedure (right). (**C**) TEE images showing a reasonable coaptation gap (0.6 cm) on the left vs. a large coaptation gap (1.5 cm) between posterior and septal leaflets. The latter is an unfavorable feature for transcatheter edge-to-edge repair techniques. (**D**) 3D TEE image demonstrating an ICD across the TV impinging on the septal leaflet resulting in displacement of the leaflet and lack of coaptation in the systole. Color Doppler image on the right shows torrential TR with a wide base extending from anteroseptal to posteroseptal commissure.

Variations exist in the healthy population. First reported by Hahn et al. (10), only 54% of tricuspid valves consist of the trileaflet configuration, while the rest have two, four, or more leaflets (Figure 1A). A septal papillary muscle may be absent in a quarter of the population (9–11). These varying configurations add to the already inherent architectural complexity of the TV structure, and make the TV highly sensitive to annular dilation, which then causes leaflet malcoaptation such that either the central or eccentric regurgitation jets can occur, of varying severity (11). The heterogeneity of the entire valve may also have implications for optimal treatment strategies in the future.

Tricuspid regurgitation etiologies have been classified into primary and secondary causes (12). Primary, inherent disorders of the valve include congenital abnormalities such as Ebstein anomaly, myxomatous degeneration, rheumatic heart disease, infective endocarditis, and traumatic injury due to blunt chest trauma (9, 11, 12). Primary TR can also occur post heart transplant as valvular degeneration due to cardiac allograft vasculopathy or be iatrogenic due to repeated heart biopsies causing leaflet perforation and chordal damage (12, 13).

Secondary, functional TR is a consequence of surrounding pathology within the heart whereby abnormal atrial or ventricular remodeling causes annular dilation and leaflet tethering. Cardiomyopathies leading to reduced ventricular function, or impaired mitral or aortic valve function, can all have deleterious bearing on the TV (9). Advances have been made to further subclassify secondary TR. Atrial functional TR occurs in the presence of the preserved left ventricular (LV) function, when there is right atrial enlargement, basal right ventricular (RV) remodeling, and dilation, which causes TV annular dilation pulling the leaflets apart such that the coaptation and tenting height are reduced (14–16). Ventricular functional TR occurs in the setting of left-sided heart disease, pulmonary hypertension, and intracardiac shunting, whereby RV dilation throughout the entire chamber causes increased leaflet tethering more so than annular dilation, resulting in increased tenting height and lack of coaptation (14, 17).

Recently, a new classification has been proposed to include cardiac implantable electronic device (CIED) related-regurgitation as a separate entity, as TR caused by pacemakers and defibrillators lead to interaction with leaflets, and the subvalvular apparatus is not an intrinsic leaflet abnormality (18).

## Assessment and quantification of severity

Echocardiography remains the diagnostic standard in evaluating TR, with transthoracic echocardiogram (TTE) and transesophageal echocardiogram (TEE) imaging necessary to characterize valve anatomy and regurgitation. No single measurement best defines TR severity, and as such, guidelines recommend a multiparametric approach using several qualitative, semiquantitative, and quantitative parameters incorporated together (6, 12, 19). To further guide transcatheter-based therapies and qualify subsequent reductions in TR severity, a five-grade regurgitation scheme was set forth by Hahn and Zamorano (20) expanding the severity grading to include massive and torrential grades.

Structural valve assessment is made by visualizing the leaflet morphology and identifying the presence of intrinsic leaflet pathologies such as flail, prolapse or perforation, or functional causes with leaflet tethering, coaptation, and tenting height. Dilated right atrial (RA), RV chambers, and reduced RV function further direct TR etiology and chronicity. Continuous wave (CW) Doppler interrogation across the regurgitant jet demonstrating a triangular-shaped, early-peaking waveform indicates rapid pressure equalization between the RA and RV, and is a sign of significant TR. Color flow jet area can be used; however, it often underestimates TR severity in the presence of low flow velocities and dilated RA (6).

Semiquantitative measurements in TR severity include the vena contracta (VC), vena contracta area (VCA), proximal isovelocity surface area (PISA), and hepatic vein flow evaluation. While VC is a simple and reproducible parameter to quantify TR, depending on echocardiographic view, the regurgitant jet could be under- or overestimated. PISA is used to calculate the quantitative parameters of the effective regurgitant orifice area (EROA) and regurgitant volume (RVol) (6). However, PISA underestimates TR severity in 20%–50% of patients for several reasons (6, 21, 22). The low-pressure, low-velocity system of the right heart can reduce PISA radius. The shape of the TR jet does not form a hemispherical flow convergence, underestimating the true severity of the leak. Regurgitation is also a dynamic process that varies throughout systole, therefore a PISA measurement at a fixed point during the cycle is not representative of the entire jet (21). Emerging data have shown that reformulating the PISA equation by incorporating the angle of the tethered leaflets, as well as accounting for the low regurgitant flow velocities, improved subsequent EROA and RVol calculations and resulted in reclassifying TR severity grade in 37% of the studied patients (21). The presence of hepatic vein systolic flow reversal is a specific sign supporting severe or greater tricuspid regurgitation (23).

Vena contracta area remains the best method to quantify TR severity and the feasibility of obtaining an accurate measurement has become more attainable with advances in 3D echocardiography and multiplanar imaging (24). As regurgitant jets often have complex shapes, VCA can accurately be measured at the level of the regurgitant orifice. Careful consideration must be taken as this may also underestimate the jet severity given that the jet area rarely occurs in a single plane (24). Exact cutoffs for 3D VCA have yet to be determined (6, 19, 25). The use of Doppler hemodynamics [the difference between tricuspid valve and left ventricular outflow tract (LVOT) or right ventricular outflow tract (RVOT) flow] to calculate EROA and regurgitant volume is another potentially valuable method to quantify TR that is currently under investigation.

Tricuspid coaptation gap (TCG) has also been studied as a marker of grading TR severity as a means to define greater than severe functional TR (26). The authors found that a TCG cutoff of 10 mm had the best combination of sensitivity and specificity, at 83% and 100%, respectively, for predicting very severe TR (VSTR), with better measurement reproducibility compared with two-dimensional EROA (26). EROA correlated poorly with TCG in patients with at least severe TR because of inherent PISA limitations; therefore, TCG may be an important and reproducible quantification parameter to grade greater than severe TR.

With the advent of cardiac CT and MRI imaging, further qualitative and quantitative characterizations can be made. Cardiac MRI remains the preferred method to assess RV function and provides accurate quantification of right ventricular stroke volume (SV) to obtain regurgitant volume and regurgitant fraction (RF) (6, 19). MRI also evaluates for atrial and ventricular remodeling. Cardiac MRI is a valuable tool when there is discordance between echocardiographic findings and clinical presentation (19). Although quantification is not possible with cardiac CT, it provides an additional modality for accurate preoperative planning for intervention (27).

## Preprocedural imaging—assessment of TR morphology

Following accurate assessment and grading of TR severity, the etiology of regurgitation as well as anatomic configuration must be defined to guide transcatheter treatment strategies. TEE is the modality of choice for this stage, allowing primary vs. secondary TR to be delineated, leaflet arrangement to be specified, and several important parameters necessary for intervention such as leaflet length, coaptation gap, and tethering to be assessed.

In secondary TR, the underlying pathophysiological mechanism should first be treated and optimized hemodynamically, and the TV be reassessed, prior to intervention (28). While transcatheter treatment outcomes data are actively under study, there is a suggestion of increased residual TR risk following intervention in patients with a four-leaflet configuration (29). A central, anteroseptal regurgitant jet and coaptation gap ≤7 mm have been associated with procedural success, while increased EROA and valve tenting are associated with procedural failure (30).

A certain leaflet length is needed for proper grasping and to avoid the risk of single leaflet device attachment. Two- and three-dimensional (3D) TEE views can be used to accurately assess leaflet length and choose proper device type (Figure 1B). Measuring the coaptation gap can guide device choice and suggest procedural success (28, 30) (Figure 1C). Leaflet tethering is a common finding in ventricular functional cases because of RV dilation and septal leaflet retraction. It is important to recognize this finding as grasping tethered leaflets using edge-to-edge repair devices could be challenging, and excess tension could lead to leaflet tear.

The location of a regurgitant jet is best defined by TEE and in the transgastric view (or 3D multiplanar reformatted images), where en face view of the valve can be obtained. The jet emanating from the anteroseptal commissure is easier to treat with transcatheter edge-to-edge repair (TEER) as the anterior leaflet is generally larger. In fact, in the feasibility study on TRILUMINATE trial (31), 77% of the treated patients received clips on the anterior and septal leaflets, followed by 20% over the posterior and septal leaflets. Stabilizing the anterior or posterior leaflets to the septal leaflet is important as the tricuspid annulus dilates laterally, pushing the anterior and posterior leaflets away from the septum.

The presence of hardware such as annuloplasty bands, bioprosthetic valves, permanent pacemaker (PPM), or implantable cardioverter defibrillation (ICD) must also be identified (Figure 1D) as these can adhere, perforate, or restrict leaflet motion, all of which pose technical challenges during treatment intervention (32). The location of the lead across the TV, its mobility and relation to the leaflet, and subvalvular apparatus are all important for TEER. A posteriorly located lead, within the posteroseptal commissure, is ideal for repair using the edge-to-edge technique. A lead located in the middle of the valve or one that is mobile can make grasping the leaflets difficult owing to its interaction with the device delivery system. Furthermore, the closer the lead is to the jet location, the greater the attenuation artifact overlaying the leaflets. Leaflet impingement by leads, or involvement of the subvalvular apparatus, can impact proper leaflet coaptation and result in varying degrees of TR. These cases are difficult to treat using TEER techniques, as the leaflets are pulled away from the coaptation line by the leads, and even successful approximation of the remaining leaflets may not overcome the pulling impact of the leads. Hence, the likelihood of residual TR in such cases remains high. In addition, although a common etiology of regurgitation, leads could also be unrelated to TR development with the origin of the regurgitant jet being distant from the lead.

Imaging of the TV in the presence of hardware therefore is especially important and can pose its own technical challenges. TEE and 3D image reconstruction, along with multiplanar imaging, allow for the anatomy to be imaged on both the atrial and ventricular sides, visualize lead trajectory, and leaflet motion (32). Such information can best be acquired by the transgastric view or 3D volume-rendered equivalent (30). Intracardiac echocardiography (ICE) during transcatheter therapies can be a complement to TEE as it allows for closer imaging of the TV and its annular plane with similar temporal resolution, understanding however that the limited depth of the imaging, limited 3D field view, cost, and bleeding risk associated with venous vascular access are notable limitations to consider (28, 33).

## Current treatment strategies

**Figure 2:**
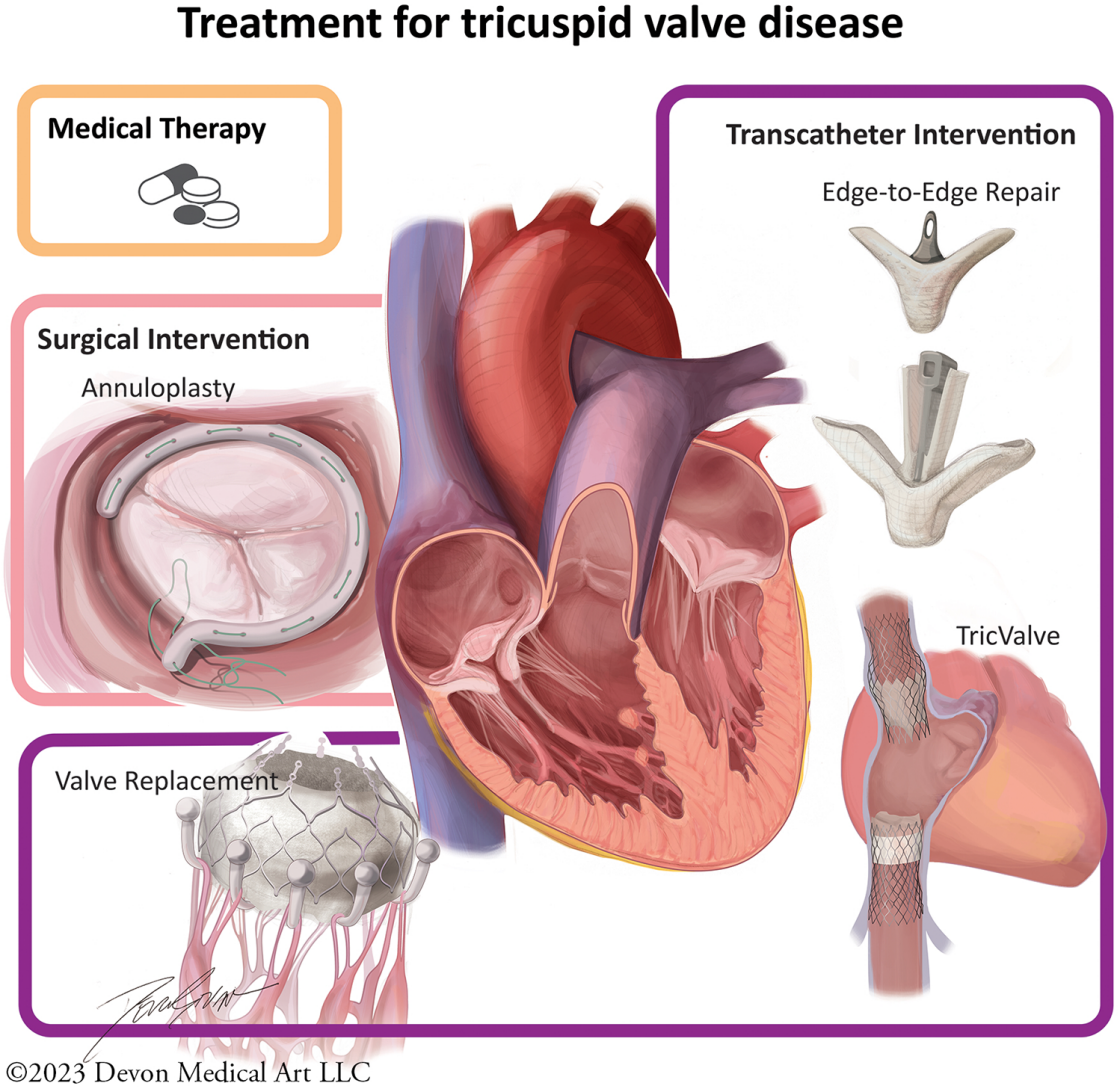
Tricuspid valve intervention strategies. Medical therapy is limited to diuretics and mineralocorticoid receptor 5 antagonists. Surgical techniques involve annuloplasty ring or valve replacement. Several percutaneous devices are currently being investigated.

The current management strategies involve a combination of medical and invasive approaches (Figure 2). Medical strategies are limited, and principally involve diuretic therapy to treat the sequella of right-sided heart failure, in combination with treating the underlying pathology in secondary TR (17). Mineralocorticoid receptor 5 antagonists (MRA) may also be used to attenuate inappropriate renin-angiotensin-aldosterone system activation in heart failure (6). In patients with atrial fibrillation, rhythm control can mitigate annular dilation and improve TR (6). For those with concomitant mitral regurgitation, transcatheter therapies with edge-to-edge repair have shown to reduce TR in more than a third of patients (34).

Surgical intervention strategies and timing depend on etiology of TR and are nuanced. Surgery is often recommended in symptomatic patients with severe TR of primary etiology, according to the 2021 ESC/EACTS valvular guidelines (6). The 2020 ACC/AHA Valvular guidelines define this as a class II recommendation, while tricuspid surgical intervention at the time of concomitant left-sided valve surgery is a class I recommendation (17). Intervention for isolated severe TR, particularly in the presence of pulmonary hypertension, dilated cardiomyopathy, or previous tricuspid surgery, carries a high operative mortality rate, which has been reported between 10% and 15% (17, 35, 36). The high mortality rate may help underscore the importance of early surgical referral with the intent of seeking correction before onset of pathological RV remodeling, and liberal surgical intervention at the time of left-sided surgery (37). Asymptomatic or minimally symptomatic patients with evidence of early RV dilation or reduced RV function on echocardiography should be considered for intervention, although exact thresholds have not yet been defined (6, 19). An annuloplasty ring is preferred over valve replacement and is associated with improved event-free survival (6, 38).

## Discussion: evolving percutaneous strategies and future developments

Transcatheter approaches to TR are a growing interest, as early data demonstrate the effectiveness of reducing regurgitation, particularly in the high surgical risk population (6, 16, 28, 31). Several devices have been introduced (Figure 2) and tested in preclinical settings, and few devices are currently under study: TriClip (Abbott, Chicago, IL, USA), PASCAL (Edwards, Washington, DC, USA), TricValve (Products + Features, Vienna, Austria), and Cardioband (Edwards, Washington, DC, USA), each addressing a different TR mechanism (27, 28). Further research is currently being explored to assess improved mortality rates in high-risk patients using these techniques. Earlier referral and consideration of both transcatheter and surgical intervention before onset of RV dysfunction or presence of end-organ damage may lead to improvement in outcomes (19).

In the 1-year outcomes data of the TRILUMINATE Pivotal Trial, tricuspid regurgitation severity reduced by at least one grade in 87% of the enrolled patients at 30 days (31, 39). The TEER intervention group was also favored over the medical therapy control group, in achieving the primary outcome consisting of a hierarchical composite of death from any cause or tricuspid valve surgery, heart failure hospitalization, and improvement in quality of life as assessed by the Kansas City Cardiomyopathy Questionnaire (KCCQ) (39), although this was driven mainly by the latter. The degree of TR reduction was related to the degree of improvement in quality of life. In its secondary outcome analysis, 98% of TEER patients remained free of major adverse events (defined as cardiovascular death, new-onset renal failure, endocarditis, and non-elective cardiovascular surgery, due to TriClip device-related adverse event) at 30-days post implantation exceeding the performance goal of 90% (39). The significance of this data is further underscored by the fact that it currently remains the only prospective, randomized controlled trial with respect to TR management (39). Similarly, the Tri.fr study is actively underway as the first randomized, multicenter, academic study aimed to evaluate whether transcatheter treatment with TriClip is superior to medical therapy with regard to secondary TR within 1 year of intervention (40).

The CLASP-TR trial evaluated the Edwards PASCAL tricuspid repair system and demonstrated low complication and high survival rates at one year as well (41). The device has a high successful implantation rate, short hospital length of stay, and is associated with greater than one grade TR reduction with only one PASCAL device (41, 42). Treatment arm patients reported improvement from NYHA functional class to classes I and II as well as improvement in the six-minute walk test and an 18-point increase in the KCCQ score (41). While these results underscore the effectiveness of the PASCAL at reducing TR, as well as supporting its safety, more long-term data are necessary to determine if such outcomes will continue and successfully translate to clinical benefit for patients (42).

The TRI-REPAIR study, in which the transcatheter annuloplasty approach is explored in patients with at least moderate functional TR, showed a reduction in septolateral annular diameter, with patient improvement in 6-minute walk score and KCCQ score (43), with favorable survival and low rehospitalization rates (44). The TRICUS-EURO study whereby the TricValve system, consisting of bicaval prosthetic valves implanted in the vena cavae, was associated in significant NYHA class symptom and quality of life improvement at 6 months (38).

Diagnosis and treatment of TR remains a rapidly evolving topic, because of the complexity of the tricuspid anatomy, the challenges in identifying the etiology of regurgitation, and the heterogeneity in treatment pathways. Rapid advancements in echocardiographic techniques with 3D imaging, as well as supplementation with cardiac CT and MRI, open avenues for improved diagnosis and characterization. Transcatheter techniques currently under study will provide future treatment options with the intent to improve quality of life and reduce the mortality of the affected patient populations.
